# MINOCA: current perspectives

**DOI:** 10.18632/aging.101618

**Published:** 2018-10-26

**Authors:** Rocco A. Montone, Michele Russo, Giampaolo Niccoli

**Affiliations:** 1 Department of Cardiovascular and Thoracic Sciences, Catholic University of the Sacred Heart, Rome, Italy

**Keywords:** MINOCA, myocardial infarction, provocative test, precision medicine

Myocardial infarction (MI) with no obstructive coronary arteries (MINOCA) is a syndrome with different causes, characterized by clinical evidence of MI with normal or near-normal coronary arteries on angiography (stenosis <50%) [1,2]. Recent data in a contemporary cohort of patients with MI reported a prevalence around 10% of cases which appears to reflect daily clinical practice [3]. However, the prognosis of patients presenting with MINOCA is not as benign as reported by early cohort studies and as commonly assumed by physicians. Indeed, recent retrospective analysis of patients enrolled in the ACUITY trial showed that, compared with non-ST elevation MI patients and obstructive coronary arteries, patients with MINOCA had a higher adjusted risk of mortality at 1 year (5.2 vs. 1.6%; HR 3.44, CI 1.05–11.28) [3].

MINOCA patients represent a conundrum given the many possible aetiologies and pathogenic mechanisms associated with this syndrome [1]. For this reason, the key principle in the management of this syndrome is to clarify the underlying individual mechanisms in order to achieve patient-specific treatments. Of importance, mechanisms underlying MINOCA have been summarized in epicardial (epicardial coronary spasm or unstable coronary plaques not revealed by angiography) or microvascular (Takotsubo syndrome, myocarditis, microvascular coronary spasm, coronary embolization) [1].

**Figure 1:**
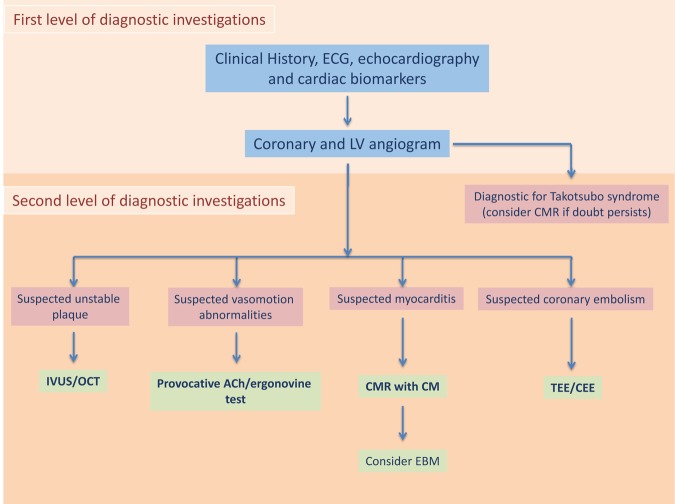
**Diagnostic flow-chart for patients presenting with MINOCA.** CEE: contrast enhanced echocardiography; CM: contrast medium; CMR: cardiac magnetic resonance; EMB: endomyocardial biopsy; IVUS: Intravascular ultrasound; OCT: optical choerence tomography; TEE: transesophageal echocardiography.

Clinical history, electrocardiogram (ECG), cardiac enzymes, echocardiography, coronary angiography and left ventricular (LV) angiography, represent the first level of diagnostic investigations to identify the causes of MINOCA [1] (Figure 1). However, more specific tests are often needed in order to discover less apparent causes of MINOCA. In particular, assessment of coronary vasomotion by intracoronary acetylcholine or ergonovine testing should be considered in order to rule out epicardial or microvascular coronary spasm when, based on clinical presentation, the presence of coronary vasomotor abnormalities is suspected [4] (Figure 1). Indeed, the identification of functional alterations in the setting of MINOCA is clinically relevant. Accordingly, we have recently demonstrated that in patients presenting with MINOCA and suspected coronary vasomotor abnormalities, intracoronary provocative test with either ergonovine or acetylcholine was positive without any complication in nearly half of patients. Epicardial spasm was detected in 24 (64.9%) patients and microvascular spasm in 13 (35.1%) patients. Patients with a positive test had a significantly higher occurrence of death from any cause, cardiac death and readmission for acute coronary syndromes, thus identifying a high-risk subset of MINOCA patients [5]. Importantly the only predictor of a worse outcome was interruption of treatment with calcium antagonists during follow up. At the same time, patients with multiple cardiovascular risk factors and subcritical coronary stenoses at angiography may require further investigation with an intravascular imaging technique in order to detect the presence of unstable plaques. Of importance, from a therapeutic point of view, the identification of a functional alteration of coronary circulation should prompt the use of vasodilators and in particular calcium channel blockers, while the identification of a non-obstructive unstable plaque should prompt an intensive control of cardiovascular risk factors and a dual antiplatelet treatment [6]. Finally, cardiac magnetic resonance imaging may help to identify patients with myocarditis or Takotsubo syndrome as cause of MINOCA.

In conclusion, the key principle in the management of patients presenting with MINOCA is to clarify the underlying individual mechanisms in order to achieve patient-specific treatments, according to a precision medicine approach.
